# Enhanced Recovery After Surgery (ERAS®) protocol in patients undergoing laparoscopic resection for stage IV colorectal cancer

**DOI:** 10.1186/s12957-015-0745-9

**Published:** 2015-12-04

**Authors:** Michał Pędziwiatr, Magdalena Pisarska, Michał Kisielewski, Piotr Major, Maciej Matłok, Mateusz Wierdak, Michał Natkaniec, Andrzej Budzyński

**Affiliations:** 2nd Department of General Surgery, Jagiellonian University Medical College, Kopernika 21, 31-501 Kraków, Poland; Department of Endoscopic, Metabolic and Soft Tissue Tumors Surgery, Jagiellonian University Medical College, Kopernika 21, 31-501 Kraków, Poland

**Keywords:** Fast-track surgery, Colorectal cancer, Laparoscopy, Enhanced recovery after surgery, Perioperative care

## Abstract

**Background:**

There is strong evidence for the use of Enhanced Recovery After Surgery (ERAS) protocol with colorectal surgery. However, in most studies on ERAS, patients with stage IV colorectal cancer (CRC) are commonly excluded. It is not certain if the ERAS protocol combined with laparoscopy improves outcomes in this group of patients as well.

The aim of the study is to assess the feasibility of the ERAS protocol implementation in patients operated laparoscopically due to stage IV CRC.

**Methods:**

A prospective analysis of patients undergoing laparoscopic colorectal surgery was performed. Group 1 included patients with stages I–III, and group 2 included patients with stage IV CRC. Demographic, surgical factors, length of stay (LOS), complications, readmissions, ERAS implementation and early postoperative recovery were compared between the groups.

**Results:**

Group 1 included 168 patients, and group 2 included 20 patients. There was no difference in the age, sex, BMI, ASA, cancer localisation or surgical parameters. No statistically significant difference was noted in complications (26.8 vs 20 %, *p* = 0.51344), LOS (4.7 vs 5.7 days, *p* = 0.28228) or readmissions (6 vs 10 %, *p* = 0.48392). The ERAS protocol compliance was 86.3 and 83.0 %, respectively (*p* = 0.17158).

**Conclusions:**

Implementation of the ERAS protocol and laparoscopic surgery among patients with stage IV CRC is feasible and provides similar short-term clinical outcomes and recovery as with patients with stages I–III.

## Background

Over the last 20 years, the perioperative care has improved significantly due to the better understanding of pathophysiological mechanisms underlying stress response to surgery. It was Kehlet who first observed that minimally invasive surgery together with appropriate analgesia, early mobilisation and oral feeding resulted in better outcomes [1]. Although initially the main goal of the so called fast-track surgery was to shorten the length of stay, the most recent concept is more complex—it aims to attenuate stress response and decrease the negative influence of insulin resistance in postoperative period [2, 3]. This in turn results in rapid functional recovery which, according to many, is considered the most important target of the modern perioperative care [4]. The novel Enhanced Recovery After Surgery (ERAS) philosophy uses multimodal interventions such as the following: preoperative counselling, no mechanical bowel preparation, shortening preoperative fasting, balanced fluid therapy, use of laparoscopy and short-acting anaesthetic agents, appropriate pain control and early oral feeding and mobilisation [5, 6]. In contrast to traditional care, ERAS involves multidisciplinary teams of surgeons, anaesthetists, nurses, dieticians and physiotherapists. Interestingly, it is the patient who is actively involved in the treatment process and plays a key role in it [7]. There is strong evidence for the use of the ERAS protocol with colorectal cancer surgery [8, 9]. It is also well-known that adherence to the protocol correlates with clinical outcomes [10, 11]. However, until now, none of the studies evaluated the feasibility and effects of the ERAS protocol implementation in patients with colorectal cancer (CRC) stage IV, according to the American Joint Committee on Cancer (AJCC), compared with patients with stages I–III. In most ERAS-related articles, staging of cancer is not taken into account. Moreover, stage IV is a common exclusion criterion from the study [12–14]. It might be important in the context of previously conducted research in the field of open colorectal surgery, with traditional perioperative care, where the stage of disease was found to influence the clinical outcomes [15]. However, it is unknown whether these findings can be simply transferred to the groups of patients operated laparoscopically and whose perioperative care was based on the ERAS protocol. Establishing the possibility of ERAS implementation and analysing its influence on clinical outcomes can be crucial for this particular group of patients.

The aim of the study was to assess the feasibility and effects of the ERAS protocol implementation in patients with stage IV CRC.

## Methods

Prospective analysis was conducted on 188 consecutive patients undergoing laparoscopic colorectal resection from January 2012 till February 2015. Inclusion criteria were as follows: age >18 years, elective laparoscopic surgery due to histologically confirmed CRC and perioperative care based on ERAS protocol principles. Patients submitted to open or emergency surgery, with concomitant inflammatory bowel diseases, or patients whose perioperative care according to ERAS protocol was not possible (for instance due to an immediate postoperative course continued on an intensive care unit), as well as patients with rectal cancer operated with endoscopic techniques like transanal endoscopic microsurgery (TEM) or transanal total mesorectal excision (TaTME), were excluded from the study.

A 16-element ERAS protocol was introduced in our unit in 2012 (Table 1). Mean compliance in patients with CRC at the present moment is near 85 %.

**Table 1:** ERAS protocol used in our unit

1. Preoperative counselling and patient’s education	
2. No bowel preparation (oral lavage in the case of low rectal resection with TME and defunctioning loop ileostomy)	
3. Preoperative carbohydrate loading (400 ml of Nutricia preOp® 2 h prior surgery)	
4. Antithrombotic prophylaxis (Clexane® 40 mg sc. starting in the evening prior surgery)	
5. Antibiotic prophylaxis (preoperative cefuroxime 1.5 g + metronidazole 0.5 g i.v. 30–60 min prior surgery)	
6. Laparoscopic surgery	
7. Balanced intravenous fluid therapy (<2500 ml intravenous fluids during the day of surgery, less than 150 mmol sodium)	
8. No nasogastric tubes postoperatively	
9. No drains left routinely for colonic resections, one drain placed for <24 h in case of TME	
10. TAP (transversus abdominis plane) block, epidural anaesthesia in cases with high risk of conversion	
11. Avoiding opioids, multimodal analgesia (oral when possible—paracetamol 4 × 1 g, ibuprofen 2 × 200 mg, metamizole 2 × 2.5 g, or ketoprofen 2 × 100 mg)	
12. Prevention of postoperative nausea and vomiting (PONV) (dexamethasone 8 mg i.v., ondansetron 8 mg i.v., metoclopramide 10 mg i.v.)	
13. Postoperative oxygenation therapy (4–6 l/min.)	
14. Early oral feeding (oral nutritional supplement 4 h postoperatively, light hospital diet and oral nutritional supplements on the first postoperative day, full hospital diet in the second postoperative day)	
15. Urinary catheter removal on the first postoperative day	
16. Full mobilisation on the first postoperative day (getting out of bed, going to toilet, walking along the corridor, at least 4 h out of bed)	

Patients were divided into two groups according to the stage of cancer. Group 1 included patients whose CRC was staged I to III according to the American Joint Committee on Cancer (AJCC) grading system and who underwent radical surgical treatment. Group 2 included patients with stage IV CRC, patients with distant resectable metastases who underwent colorectal resection as part of a two-step surgical treatment (intention to treat) as well as those with disseminated CRC who underwent palliative colorectal resection. In these patients, systemic chemotherapy as first-line treatment was contraindicated due to complications related to the tumour such as severe anaemia, obvious bleeding or high risk of full bowel obstruction in the near future.

**Fig. 1:**
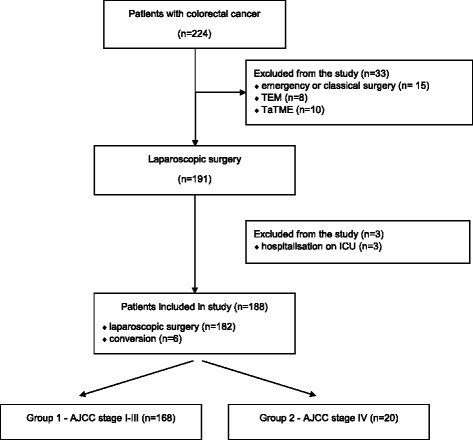
ITT flowchart

During the analysed period, 224 patients with CRC underwent operations. Fifteen patients underwent emergency or primarily open procedure and thus were excluded from the study. Similarly, 18 patients with rectal cancer who underwent TEM or TaTME were excluded from further analysis. Three patients were excluded from the study due to the necessity of immediate postoperative stay at the intensive care unit, and ERAS protocol was not applied (Fig. 1).

The age, sex, body mass index (BMI), American Society of Anaesthesiologists (ASA) scale, localisation of the cancer, intraoperative blood loss and conversion rate were compared between the two groups. The primary outcomes were the following: length of stay (LOS) in hospital, number of complications and frequency of readmissions during 30 days after discharge. The secondary outcome was compliance with the ERAS protocol. The tertiary outcome was the level of recovery after operation.

Postoperative complications are presented according to the 5-grade Clavien-Dindo scale [16, 17]. Compliance with the ERAS protocol was assessed by calculations of accomplishment or failure of 13 pre- and perioperative protocol elements dependent on medical stuff. The level of recovery after surgery was established basing on the passage of first stool as well as implementation of the following elements of the protocol: tolerance of oral diet on the first postoperative day, mobilisation of the patient on the day of surgery and no need for postoperative opioids consumption.

Discharge criteria were the following: full mobilisation, good tolerance of oral diet, no need for intravenous fluids or drugs and no complications. Readmission was defined as any re-hospitalisation within the first 30 days after discharge.

A demographic analysis of both groups is presented in Table 2.

**Table 2:** Demographic analysis of patient groups

Parameter	Group 1 AJCC stages I–III	Group 2 AJCC stage IV	*p* value
Number of patients, *n*	168(89.4 %)	20 (10.6 %)	–
Females, *n* (%)	81 (48.2 %)	7 (35 %)	0.26290
Males, *n* (%)	87 (51.8 %)	13 (65 %)	
Mean age, years ± SD	66.3 ± 12.8	65.9 ± 10.5	0.82724
BMI, kg/m^2^ ± SD	26.3 ± 5.1	25.5 ± 5.6	0.32776
ASA 1, *n* (%)	6 (3.6 %)	–	0.89411
ASA 2, *n* (%)	98 (58.3 %)	13 (65 %)	
ASA 3, *n* (%)	60 (35.7 %)	7 (35 %)	
ASA 4, *n* (%)	4 (2.4 %)	–	
Colon, *n* (%)	119 (70.8 %)	13 (65 %)	0.58972
Rectum, *n* (%)	49 (29.2 %)	7 (35 %)	
AJCC stage I, *n* (%)	65 (38.7 %)	–	–
AJCC stage II, *n* (%)	60 (35.7 %)	–	
AJCC stage III, *n* (%)	43 (25.6 %)	–	
AJCC stage IV, potentially resectable, *n* (%)	–	5 (25 %)	
AJCC stage IV, palliative resection, *n* (%)	–	15 (75 %)	
Right hemicolectomy, *n* (%)	62 (36.9 %)	5 (25 %)	–
Left hemicolectomy, *n* (%)	12 (7.1 %)	–	
Sigmoid resection, *n* (%)	45 (26.8 %)	6 (30 %)	
Total mesorectal excision, *n* (%)	48 (28.6 %)	4 (20 %)	
Hartmann’s operation, *n* (%)	–	4 (20 %)	
Abdominoperineal excision, *n* (%)	1 (0.6 %)	1 (5 %)	

The study obtained the ethical approval from the local ethics review committee of the Jagiellonian University, Krakow (approval number: KBET/53/B/2014) and has been performed in accordance with the ethical standards laid down in the 1964 Declaration of Helsinki and its later amendments. Informed consent was obtained from all patients before surgery.

Statistical analysis was performed with StatSoft STATISTICA v.10. Tests were selected depending on the type of the variables. For the qualitative variable test, chi-square was used. In cases of quantitative variables where no normal distribution was observed, we used the Mann-Whitney test. For calculations of ordinal variables, as ASA grade or postoperative complications assessed by the Clavien-Dindo classification, we also used the Mann-Whitney test. *p* < 0.05 was chosen as statistically significant. For calculations of mean LOS in hospital, patients with extreme values (>3 IQR—>20 days, 5 patients—four from group 1 and one from group 2) were excluded.

## Results

No statistically significant difference between groups was observed for age, sex, BMI, ASA scale, localisation of the tumour and type of surgery performed. The groups were not significantly different in the matter of operative time, intraoperative blood loss and conversion rate (Table 3).

**Table 3:** Intraoperative parameters in analysed groups

Parameter	Group 1 AJCC stages I–III	Group 2 AJCC stage IV	*p* value
Mean operative time, min. ± SD	190.7 ± 60.6	189.8 ± 64.3	0.88045
Median operative time, min (IQR)	180 (140–230)	180 (147–225)	
Mean intraoperative blood loss, ml ± SD	89.2 ± 76.1	120 ± 115.2	0.16003
Median intraoperative blood loss, ml (IQR)	50 (50–100)	100 (50–125)	
Conversion, *n* (%)	5 (3.0 %)	1 (5.3 %)	0.62643

In 45 patients (26.8 %) from group 1 and four patients (20 %) from group 2, postoperative complications occurred (*p* = 0.51344). Although mean and median LOS was different between groups, it did not reach the statistically significant difference (4.7 ± 2.9 days vs 5.7 ± 3.3 days, median 4 vs. 5 days, *p* = 0.28228). Readmissions concerned ten patients from group 1 and two patients from group 2 (6 vs 10 %, *p* = 0.48392). One patient from group 1 died in the postoperative period. Primary outcomes are presented in Table 4.

**Table 4:** Postoperative outcomes in analysed groups

Parameter	Group 1 AJCC stages I–III	Group 2 AJCC stage IV	*p* value
Patients without complications, *n* (%)	123/168 (73.2 %)	16/20 (80 %)	0.51344
Patients with complications, *n* (%)	45/168 (26.8 %)	4/20 (20 %)	
Clavien-Dindo 1, *n* (%)	28/168 (16.7 %)	–	0.75389
Clavien-Dindo 2, *n* (%)	6/168 (3.5 %)	1/20 (5 %)	
Clavien-Dindo 3, *n* (%)	10/168 (6 %)	3/20 (15 %)	
Clavien-Dindo 5, *n* (%)	1/168 (0.6 %)	–	
Mean length of hospital stay, days ± SD	4.7 ± 2.9	5.7 ± 3.3	0.28228
Median length of hospital stay, days (IQR)	4 (3–6)	5 (3–8)	
Readmission, *n* (%)	10 (6 %)	2 (10 %)	0.48392
Mortality within 30 days post-surgery, *n* (%)	1 (0.6 %)	–	–

No statistically significant difference between groups was noted in terms of compliance to the pre- and intraoperative ERAS protocol elements (86.3 ± 13.0 % vs 83.0 ± 11.3 %, *p* = 0.17158). When separate elements were analysed, only early urinary bladder catheter removal (<24 h postoperatively, 86.3 vs 60 %, *p* = 0.00272) and peritoneal drainage (used in cases of colonic surgery or >24 h in cases of TME, 76.8 vs. 45 %, *p* = 0.00236) were different. The remaining elements did not differ in a statistically significant manner. Regarding early postoperative recovery, our observation also did not show any statistically significant difference between the groups in passage of first stool, opioid use, tolerance of oral diet and mobilisation in the first postoperative day (Table 5).

**Table 5:** Compliance with perioperative parameters in analysed groups

Parameter	Group 1 AJCC stages I–III	Group 2 AJCC stage IV	*p* value
Selective mechanical bowel preparation	114 (68 %)	16 (80 %)	0.51522
Preoperative CHO-loading	126 (75 %)	16 (80 %)	0.62294
Balanced fluid therapy	139 (82.7 %)	14 (70 %)	0.16652
Urinary catheterisation after surgery <24 h	145 (86.3 %)	12 (60 %)	0.00272
Selective peritoneal drainage	129 (76.8 %)	9 (45 %)	0.00236
Compliance with ERAS protocol, % ± SD	86.3 ± 13.0	83.0 ± 11.3	0.17158
Functional postoperative recovery			
Tolerance of full oral diet in the first postoperative day	121 (72 %)	12 (60 %)	0.26385
Full mobilisation on the first postoperative day	147 (87.5 %)	15 (75 %)	0.12582
No need for opioids	106 (63.1 %)	15 (75 %)	0.29339
Passage of first stool, (days, mean, ±SD, median)	(1–4 days), 2.29 ± 1.13 days, median 2 days	(0–7 days), 2.23 ± 1.47 days, median 2 days	0.71564

The table presents only selected ERAS protocol elements in which compliance was lower than 95 %

## Discussion

In this study involving patients operated laparoscopically due to CRC, where perioperative care was based on ERAS protocol principles, we discovered that complications, prolonged LOS and readmissions did not occur more often in patients with stage IV CRC than in the group of patients with less advanced stages of cancer. In both groups, compliance with the ERAS protocol was also similar. Additionally, no difference was noted in recovery parameters.

Discussions concerning whether to perform palliative resections in patients with advanced CRC are still going on. According to some authors, resection of an asymptomatic tumour can be beneficial [18–20]. Moreover, appropriately planned operations in patients with disseminated cancer can prevent such life-threatening complications as obstruction, tumour perforation or anaemia in the event of chronic bleeding [21, 22]. On the other hand, opponents of such an approach underline the fact that this kind of treatment of patients with advanced CRC does not prolong survival [23]. According to EURECCA colorectal consensus conference, the primary treatment option in the case of disseminated cancer should be systemic chemotherapy [24]. There is an opinion that advanced cancer predisposes to complications like anastomotic leakage or adverse cardiovascular events [25–27]. It should be emphasised that patients with distant metastases in our study group were operated on either with intention to cure (potentially resectable metastases) or in the situation of complications related to cancer (chronic anaemia, visible bleeding, obstructing cancer that might lead to full mechanical obstruction in the near future). These patients were disqualified from systemic chemotherapy, and surgery remained the only possible modality. Our study, however, did not confirm the hypothesis of worse outcomes in such patients. There are several possible explanations for that. First of all, practically all surgeries were performed laparoscopically, which is generally associated with a reduced number of complications [28, 29]. Moreover, the number of complications in our study is comparable, if not smaller, with results presented by other centres [30]. It may be explained by the combination of laparoscopy with the ERAS protocol. Such synergistic effect is known to decrease the risk of complications and improve recovery [9, 14, 31].

The majority of publications about the ERAS protocol and laparoscopy concerns patients with stages I–III CRC. Available data about patients with stage IV CRC is sparse, as commonly disseminated cancer is a usual criterion of exclusion from the study [12–14]. What is worth noting is that we did not observe inferior implementation of ERAS protocol in patients with advanced cancer. Compliance to ERAS protocol was over 80 % in both groups. This confirms the possibility of the use of ERAS in patients with any stage of cancer. ERAS implementation allowed patients to reach comparable recovery, measured by the ability to pass stool, tolerate oral diet, mobilisation in first postoperative day and the need for opioids.

Our study has certain limitations typical for single-centre studies. An additional limitation is the relatively small number of patients recruited to the group with stage IV CRC, resulting in different group sizes. This can create a risk of type II error and requires further analysis based on a larger group of patients. Moreover, we did not assess long-term results in both groups. This is the subject of our future studies.

## Conclusions

A combination of laparoscopy with the ERAS protocol provides similar short-term outcomes in patients with stage IV CRC. This can be an argument when deciding whether to perform palliative resection when surgery in this group of patients is necessary. Due to the abovementioned limitations, further research on larger groups is required.

## Abbreviations

AJCC: American Joint Committee on Cancer
BMI: body mass index
CRC: colorectal cancer
ERAS: Enhanced Recovery After Surgery
LOS: length of stay
TAP block: transversus abdominis plane block

## References

[1] Bardram L, Funch-Jensen P, Jensen P, Crawford ME, Kehlet H (1995). Recovery after laparoscopic colonic surgery with epidural analgesia, and early oral nutrition and mobilisation. Lancet.

[2] Kehlet H, Wilmore DW (2002). Multimodal strategies to improve surgical outcome. Am J Surg.

[3] Scott MJ, Baldini G, Fearon KCH, Feldheiser A, Feldman LS, Gan TJ (2015). Enhanced Recovery After Surgery (ERAS) for gastrointestinal surgery, part 1: pathophysiological considerations. Acta Anaesthesiol Scand.

[4] Aahlin EK, von Meyenfeldt M, Dejong CH, Ljungqvist O, Fearon KC, Lobo DN (2014). Functional recovery is considered the most important target: a survey of dedicated professionals. Perioper Med (Lond).

[5] Nygren J, Thacker J, Carli F, Fearon KCH, Norderval S, Lobo DN (2012). Guidelines for perioperative care in elective rectal/pelvic surgery: Enhanced Recovery After Surgery (ERAS®) Society recommendations. World J Surg.

[6] Gustafsson UO, Scott MJ, Schwenk W, Demartines N, Roulin D, Francis N (2013). Guidelines for perioperative care in elective colonic surgery: Enhanced Recovery After Surgery (ERAS(®)) Society recommendations. World J Surg.

[7] Francis N, Kennedy RH, Ljungqvist O, Mythen MG (2012). Manual of fast track recovery for colorectal surgery.

[8] Greco M, Capretti G, Beretta L, Gemma M, Pecorelli N, Braga M (2013). Enhanced recovery program in colorectal surgery: a meta-analysis of randomized controlled trials. World J Surg.

[9] Vlug MS, Wind J, Hollmann MW, Ubbink DT, Cense HA, Engel AF (2011). Laparoscopy in combination with fast track multimodal management is the best perioperative strategy in patients undergoing colonic surgery: a randomized clinical trial (LAFA-study). Ann Surg.

[10] ERAS Compliance Group (2015). The impact of enhanced recovery protocol compliance on elective colorectal cancer resection: results from an international registry. Ann Surg.

[11] Gustafsson UO (2011). Adherence to the enhanced recovery after surgery protocol and outcomes after colorectal cancer surgery. Arch Surg.

[12] Feroci F, Kröning KC, Lenzi E, Moraldi L, Cantafio S, Scatizzi M (2011). Laparoscopy within a fast-track program enhances the short-term results after elective surgery for resectable colorectal cancer. Surg Endosc.

[13] Feroci F, Lenzi E, Baraghini M, Garzi A, Vannucchi A, Cantafio S (2012). Fast-track colorectal surgery: protocol adherence influences postoperative outcomes. Int J Colorectal Dis.

[14] King PM, Blazeby JM, Ewings P, Kennedy RH (2008). Detailed evaluation of functional recovery following laparoscopic or open surgery for colorectal cancer within an enhanced recovery programme. Int J Colorectal Dis.

[15] Perng D-S, Lu I-C, Shi H-Y, Lin C-W, Liu K-W, Su Y-F (2014). Incidence trends and predictors for cost and average lengths of stay in colorectal cancer surgery. World J Gastroenterol.

[16] Clavien PA, Barkun J, de Oliveira ML, Vauthey JN, Dindo D, Schulick RD (2009). The Clavien-Dindo classification of surgical complications: five-year experience. Ann Surg.

[17] Dindo D, Demartines N, Clavien P-A (2004). Classification of surgical complications: a new proposal with evaluation in a cohort of 6336 patients and results of a survey. Ann Surg.

[18] Ruo L, Gougoutas C, Paty PB, Guillem JG, Cohen AM, Wong WD (2003). Elective bowel resection for incurable stage IV colorectal cancer: prognostic variables for asymptomatic patients. ACS.

[19] Cook AD, Single R, McCahill LE (2005). Surgical resection of primary tumors in patients who present with stage IV colorectal cancer: an analysis of surveillance, epidemiology, and end results data, 1988 to 2000. Ann Surg Oncol.

[20] Konyalian VR, Rosing DK, Haukoos JS, Dixon MR, Sinow R, Bhaheetharan S (2007). The role of primary tumour resection in patients with stage IV colorectal cancer. Colorectal Dis.

[21] Joffe J, Gordon PH (1981). Palliative resection for colorectal carcinoma. Dis Colon Rectum.

[22] Rosen SA, Buell JF, Yoshida A, Kazsuba S, Hurst R, Michelassi F (2000). Initial presentation with stage IV colorectal cancer: how aggressive should we be?. Arch Surg.

[23] Cirocchi R, Trastulli S, Abraha I, Vettoretto N, Boselli C, Montedori A (2012). Non-resection versus resection for an asymptomatic primary tumour in patients with unresectable stage IV colorectal cancer. Cochrane Database Syst Rev.

[24] van de Velde CJH, Boelens PG, Borras JM, Coebergh J-W, Cervantes A, Blomqvist L, et al. EURECCA colorectal: multidisciplinary management: European consensus conference colon & rectum. 2014:50;1.e1–1.e34. Elsevier. http://www.ejcancer.com/article/S0959-8049(13)00780-6/pdf. 10.1016/j.ejca.2013.06.048 24183379

[25] Branagan G, Finnis D, Wessex Colorectal Cancer Audit Working Group (2005). Prognosis after anastomotic leakage in colorectal surgery. Dis Colon Rectum.

[26] Yun J-A, Huh JW, Park YA, Cho YB, Yun SH, Kim HC (2014). The role of palliative resection for asymptomatic primary tumor in patients with unresectable stage IV colorectal cancer. Dis Colon Rectum.

[27] Scheer MGW, Sloots CEJ, van der Wilt GJ, Ruers TJM (2008). Management of patients with asymptomatic colorectal cancer and synchronous irresectable metastases. Ann Oncol.

[28] Abraham NS, Young JM, Solomon MJ (2004). Meta-analysis of short-term outcomes after laparoscopic resection for colorectal cancer. Br J Surg.

[29] Aziz O, Constantinides V, Tekkis PP, Athanasiou T, Purkayastha S, Paraskeva P (2006). Laparoscopic versus open surgery for rectal cancer: a meta-analysis. Ann Surg Oncol.

[30] Law WL, Chu KW (2006). Outcomes of resection of stage IV rectal cancer with mesorectal excision. J Surg Oncol.

[31] Kennedy RH, Francis EA, Wharton R, Blazeby JM, Quirke P, West NP (2014). Multicenter randomized controlled trial of conventional versus laparoscopic surgery for colorectal cancer within an enhanced recovery programme: EnROL. J Clin Oncol.

